# Welfare receipt misreporting in survey data and its consequences for state dependence estimates: new insights from linked administrative and survey data

**DOI:** 10.1186/s12651-018-0250-z

**Published:** 2018-12-20

**Authors:** Kerstin Bruckmeier, Katrin Hohmeyer, Stefan Schwarz

**Affiliations:** Institute for Employment Research, Regensburger Straße 104, 90478 Nuremberg, Germany

**Keywords:** Dynamic multinomial logit model, Misreporting, State dependence, Survey data, Welfare receipt, C81, I32, I38

## Abstract

In many advanced welfare states, welfare recipients often receive benefits for long periods. This persistence of welfare receipt can be caused by two distinct mechanisms: genuine or spurious state dependence. Knowledge of which of the two mechanisms drives the observed state dependence is important because the policy implications are different. Most of the empirical evidence on state dependence relies on survey data. However, survey data on welfare receipt are subject to substantial measurement error (i.e., misreporting of welfare benefit receipt), which may also bias state dependence estimates. This paper uses rich linked survey and administrative data to measure the effect of misreporting in the survey data on the estimated state dependence in welfare receipt in Germany. We find a rate of underreporting of welfare benefits of 8.6%. Recipients with relatively good labour market chances tend to underreport benefits more frequently. Overreporting benefits is less pronounced with a rate of 1.6%. Within the survey data, we observe more transitions into and out of the welfare system. However, our estimates of state dependence in welfare receipt based on a dynamic random effects model reveal that the effect of misreporting on estimated state dependence is small, even when we distinguish between working and non-working recipients in the model.

## Introduction

Most developed countries provide basic income support for needy working-age individuals and their families. Many welfare recipients receive these benefits persistently (e.g., Bane and Ellwood 1994; Blank 1989). This persistence in welfare receipt can be explained via two distinct mechanisms (Heckman 1981a): *Genuine (or true) state dependence* arises if the experience of past welfare benefits receipt has direct consequences on future welfare receipt. This would be the case if an individual who experienced benefit receipt in the past behaves or is assessed differently in the future compared to an identical individual who did not experience benefit receipt (e.g., through the stigma of benefit receipt or a loss of human capital during unemployment). *Spurious state dependence* implies that the observed or unobserved characteristics of an individual affect the likelihood to receive welfare benefits. Welfare recipients might differ from non-recipients in socio-economic characteristics or in unobserved preferences, and individuals with disadvantageous characteristics, such as a large number of children or low educational attainment, might select themselves into welfare receipt (Königs 2014). These differences in characteristics might contribute to the different rates of future welfare receipt for welfare recipients and non-recipients.

The distinction between true and spurious state dependence is of considerable interest since policy implications are different. If state dependence is mainly driven by past welfare receipt, then policies that prevent individuals from welfare entry might decrease the persistence rates. If, in contrast, state dependence is driven by certain characteristics (such as a low educational level) of welfare recipients, then addressing these obstacles directly might be more beneficial.

Most empirical evidence on state dependence in welfare receipt relies on survey data (e.g., Cappellari and Jenkins 2008; Königs 2014; Wunder and Riphahn 2014). However, the quality of data from household surveys suffers from several problems (Meyer et al. 2015a). The problem of unit nonresponse occurs when households from the sampling frame do not answer the survey at all. The problem of item non-response arises when households participate in the survey but do not answer single questions. Both problems lead to a potential bias of survey estimates if unit and item non-response on income and wealth questions could not be considered as randomly across the population (Groves 2006; Meyer et al. 2015a; Riphahn and Serfling 2005). Last, a potential bias to survey based estimates accrues, if respondents do not answer accurately or even give false answers. Concerning welfare benefits, a substantial fraction of individuals do not report that they receive benefits (e.g., because of the stigma of benefit receipt, see, e.g., Meyer et al. 2015a). Not only this so-called underreporting can bias the estimates of state dependence when using survey data but also non-recipients who report to receive welfare benefits (overreporting). In our paper, we focus on the effects of this specific form of measurement error, under- and overreporting of welfare benefits, on the estimates of state dependence in welfare receipt. Over- and underreporting of benefits seem to be especially relevant for the analysis of state dependence if misreporting changes over time, which can lead to observed transitions between welfare receipt and non-receipt that, in reality, did not happen.

This paper uses survey data from the German panel study “Labour Market and Social Security (PASS)” for the years 2007 to 2014 to analyse the effects of benefit misreporting on estimates of state dependence. A major advantage of the PASS is that the survey data can be linked to individual administrative records from the Federal Employment Agency, which is responsible for the administration of welfare benefits in Germany. We make use of this linked data to describe the extent of misreporting in the survey data and to measure the effect of misreporting on the estimates of state dependence in welfare receipt in Germany. Germany is an interesting case to study: Although the economy managed to survive comparatively well through the last great recession, Germany is characterized by a high incidence of long-term unemployment and long-term benefit receipt. Most of the long-term unemployed receive means-tested welfare benefits called unemployment benefit II (Arbeitslosengeld II, UB II), the relevant institution for our analysis.

The welfare benefit is paid to needy households with at least one member between 15 and 64 years of age who is capable of working and whose income is insufficient to maintain the legally defined minimum income level of the household. Hence, eligibility does not depend on unemployment and a significant number of persons receive the benefits in addition to their wage income. We, therefore, focus in our empirical analysis on transitions between four different labour market states: welfare benefit receipt, welfare benefit receipt while having a job, inactivity and employment.

To model the probability of being in one of the four different labour market states, we employ a dynamic multinomial logit model with controls for observed and unobserved heterogeneity and endogenous initial conditions. We find a welfare benefit underreporting rate of 8.6% and an overreporting rate of 1.6% in the PASS data. Within the survey data, we observe slightly more transitions into and out of the welfare system. However, our estimates of state dependence in welfare receipt based on a dynamic random effects model reveal that the effect of mismeasurement caused by misreporting on estimated state dependence is small, even when we distinguish between working and non-working recipients in the model.

## Institutional background

High unemployment persistence, increasing unemployment rates and low female labour force participation in Germany gave rise to comprehensive labour market reforms in the early 2000s (see, e.g. Caliendo 2009; Eichhorst et al. 2010). One main goal of the ‘Hartz reforms’ was to increase the incentives for unemployed individuals to take up work. Hartz IV, the fourth package of the Hartz reforms, was implemented in January 2005 and intensely changed the unemployment and social assistance schemes: The previous unemployment and social assistance benefits were replaced by the means-tested welfare benefit called UB II for needy individuals who are capable of working. UB II is designed to prevent people from falling into poverty by providing a legally defined minimum income and to strengthen the incentives to take up work. Eligibility is defined at the household level, however, the relevant benefit-receiving unit is only the (core) family living in the same household (Bedarfsgemeinschaft), i.e., recipients with their partner and children aged below 25 years. When talking about households in the following, we always refer to the UB II eligible unit. Households are eligible for the welfare benefit if they pass the means test, i.e., if the household’s total needs exceed the allowable income and the household’s wealth remains below the household specific maximum. Total needs are defined by a standard benefit for each member of the household and by housing costs. The welfare benefit is paid to needy households with at least one member between 15 and 64 years of age who is capable of working. Hence, eligibility does not depend on unemployment but on at least one household member being able to work for at least 3 hours per day under regular labour market conditions.

Large groups within UB II are (a) the long-term unemployed whose claims to unemployment insurance benefits (UB I) have been exhausted, (b) the short-term unemployed who are not entitled to (sufficient) UB I, and (c) employed persons whose earnings are insufficient to maintain the legally defined minimum income level. Table 1 shows the different labour market activities of welfare benefit recipients in 2016: Of a total of approximately 6 million recipients in 2016, 4.3 million were capable of working, and the others were mainly their children. Less than half of the recipients capable of working were registered as unemployed (1.8 million). The others were participating in active labour market policy measures, employed, involved in education, engaged in child or elderly care activities, temporarily not able to work or in early retirement. The largest group among the recipients who were not registered as unemployed were employed welfare recipients working at least 15 h a week (to be not counted as unemployed). In fact, UB II acts as an in-work benefit for this group.

**Table 1:** Welfare benefit recipients and their activities 2016 (annual average)

Welfare benefit recipients and their activities		Total (in 1000)
Households		3267
Recipients		5925
Incapable of working		1613
Recipients capable of working		4312
Unemployed recipients		1777
Not unemployed recipients		2535
	*thereof*	
	Participants in labour market policy measures	495
	Employed recipients	682
	In Education	360
	Care for relatives or children	295
	Temporarily incapable of working	300
	Early retirement	162
	Else	241

Source: Statistik der Bundesagentur für Arbeit, Tabellen, Strukturen der Grundsicherung SGB II (Zeitreihe Monats- und Jahreszahlen ab 2005), Nürnberg, November 2017

In our analysis, we solely focus on UB II receipt of individuals capable of working. Because the share of employed benefit recipients is considerable and both the determinants and the patterns of state dependence could be assumed to differ between unemployed and working recipients, we additionally distinguish between working and non-working recipients in the empirical analysis.

## State dependence in welfare receipt

A small but growing number of studies aims to investigate the extent of true state dependence in welfare benefit receipt. These studies employ dynamic panel models to estimate the likelihood of receiving welfare benefit as a function of the past welfare receipt, covariates (observed heterogeneity) and time-constant individual unobserved heterogeneity.

Cappellari and Jenkins (2008) use data from the British Household Panel Survey (1991–2005) to investigate the dynamics of welfare receipt in Britain. They find that after controlling for heterogeneity, past welfare receipt is associated with a 15-percentage-point (i.e., approximately four times) higher probability of benefit receipt than if there were no benefit receipt in the last period. Hansen et al. (2006) use survey data to study social assistance dynamics in Canada from 1993 to 2000. They find evidence for the existence of true state dependence, but its extent differs among the different geographical regions of Canada. Hansen and Lofstrom (2009) use administrative panel data to study the transition dynamics among unemployment, employment and social assistance in Sweden from 1990 to 1996. They find that refugee immigrants experience a higher structural state dependence than natives. Whereas 17% of the observed state dependence for natives is structural, the same figure amounts to approximately 80% for immigrants from refugee countries. The authors argue that this observation might result from the existence of a welfare trap for refugee immigrants, while the high persistence rate of natives and non-refugee immigrants, in the raw data, mostly arises from unobserved individual heterogeneity. Bhuller et al. (2017) use comprehensive administrative data to study welfare state dependence in Norway. They find that the estimated state dependence varies strongly with the time unit of analysis: the average treatment effect of past benefit receipt increases with the level of aggregation.

Recently, the dynamics of welfare benefit receipt have gained attention in Germany. All studies are based on survey data from the Socio-Economic Panel Study (SOEP). Königs (2014) studies the dynamics of social assistance receipt in Germany before and after the ‘Hartz Reforms’ (1995–2011). He uses a broad definition of the benefit variable and finds evidence for the existence of true state dependence, although a large part of the observed persistence can be attributed to spurious state dependence through individual heterogeneity. His results imply that after controlling for individual heterogeneity a welfare recipient is 3.4 times more likely to experience welfare benefit receipt in the following year compared to a non-recipient, which results in an average partial effect of 13 percentage points. Wunder and Riphahn (2014) and Riphahn and Wunder (2016) distinguish among three labour market states: welfare, inactivity and employment. They find that the high welfare persistence rates in raw data can be largely explained by observed and unobserved characteristics. The persistence in welfare receipt for natives and immigrants is reduced from 75% and 77% to 9% and 3%, respectively, after controlling for observable characteristics and time-invariant unobserved heterogeneity (Wunder and Riphahn 2014). Königs (2014) ascribes the difference between his estimates for true state dependence and the ones of Wunder and Riphahn (2014) to the differences in the definition of the sample and of the benefit variable.

## Misreporting of welfare receipt and its impact on estimated state dependence

Most studies investigating state dependence in welfare receipt rely on survey data, which might be subject to measurement error due to the misreporting of benefit receipt. Respondents might misreport their benefit receipt for several reasons (Eggs 2016; Meyer et al. 2015b). First, individuals misremember their benefit receipt (e.g., its name or its timing). Second, they refrain from reporting their benefit receipt because of the stigma of welfare benefit receipt, the sensitivity of the information or the desire to decrease the interview burden. Different studies have shown that the underreporting of welfare receipt is more common than the overreporting of welfare receipt (see e.g. Bollinger and David 2001; Bruckmeier et al. 2014). Underreporting of benefits has been studied for different benefits in the United States. Bollinger and David (1997, 2001) use linked administrative data to study the misreporting of Food Stamp programme participation in the Survey of Income and Program Participation (SIPP). They find that Food Stamp participation is not reported by 12% of the participants in their sample. Meyer and Mittag (2018) and Meyer et al. (2015b) study misreporting for several major government benefit programmes in the US (e.g., Temporary Assistance for Needy Families (TANF), Food Stamps, unemployment insurance). Using linked administrative and survey data, Meyer and Mittag (2018) find underreporting to differ by programme and by survey, ranging between 30 and 60% of the participants not reporting their benefit receipt. Underreporting is related to both the benefit receipt and the amount of benefit received.

Meyer et al. (2015b) study underreporting of participation in different programmes by comparing administrative data to the weighted results from survey data. Comparing different programmes, they do not find evidence of the major importance of stigma and the sensitivity of information but attribute the differences in underreporting to the survey design and the desire to decrease the interview burden.

Misreporting has also recently been studied for the German welfare benefit UB II, on the basis of the panel study “Labour Market and Social Security (PASS)” linked to administrative data. Bruckmeier et al. (2014) find an underreporting rate of 10.5% among welfare recipients in Germany. Moreover, they find that welfare recipients with relatively good labour market prospects, who are young or who receive welfare benefits only for a short period are less likely to report their welfare receipt. Furthermore, the characteristics of the interviewer and of the interview matter (Bruckmeier et al. 2015). Eggs (2016) studies misreporting of welfare receipt in Germany based on a longitudinal perspective and finds that the extent of underreporting decreases over panel waves and is correlated with several other variables, such as age and household income. Consequently, misreporting cannot be considered random.

Many studies that analyse benefits to inform policy makers about programme success rely on survey data. The problem of misreporting might lead to a bias in their estimates and imply wrong advice to policy makers. However, the studies to date on the role of misreporting in survey data when analysing benefits are sparse. Looking at data from the State New York, Meyer and Mittag (2018) find that analyses based on survey data subject to underreporting lead to an underestimation of the number of participants and of poverty reduction provided through a benefit and to an overestimation of the number of disconnected people. Bollinger and David (1997, 2001) find that even a small extent of underreporting can lead to biased estimates of the participation probability in the Food Stamps programme.^1^ The majority of studies on state dependence in welfare receipt rely on survey data because suitable administrative data covering welfare recipients and non-recipients are often not available (exceptions are Bhuller et al. 2017 for Norway and Hansen and Lofstrom 2009 for Sweden). Therefore, misreporting might also affect the transition rates and estimates of state dependence in these studies. However, there is no evidence yet on the effects of misreporting on the state dependence analyses. It is not straightforward how the estimated state dependence is likely to be affected by misreporting. On the one hand, the direction of the bias depends on whether individuals continuously underreport their welfare receipt or whether they only underreport once. If the misreporting of benefit receipts decreases over time (as observed by Eggs 2016), then studies based on survey data count a number of erroneous transitions and might therefore underestimate the state dependence in welfare receipt. On the other hand, the direction of the bias depends on who misreports. As better risk recipients are more likely not to report their welfare receipt, studies based on survey data might overestimate the state dependence in welfare receipt.

## Data and method

### Data

We use data from the annual panel study “Labour Market and Social Security” (PASS). PASS was especially designed for research on unemployment and poverty dynamics (Trappmann et al. 2013). It combines a subsample of welfare recipients with a population sample that oversamples households with low socio-economic status. The subsample of welfare recipients is drawn by the use of an identification number assigned by the welfare administration. The sample is refreshed in each wave by a sample of households from welfare benefit inflows. For the population subsample, population registers compiled by the German municipalities are used.^2^ The first wave of PASS was conducted in 2006/2007 and is not considered in our analysis due to the changes in the survey instruments and interview programme after the first wave. Hence, our analysis uses data from waves 2 to 8, with 95,823 individual-year-observations (see Table 2). We only include working-age individuals (25 to 57 years^3^) who are working or available for the labour market (i.e., not in education or incapable of working) in our analysis sample. Furthermore, civil servants and self-employed are excluded, which leaves us with 54,874 observations.

**Table 2:** Sample selection

Total number of observations	114,777
Observations excluded because…	
Wave 1	18,954
Pensioners and young people < 25 years	37,698
Pupils, students, civil servants, individuals incapable of working, self-employed	3251
No consent to linking	2374
Not found in administrative data	8091
No information on UB II receipt (PASS)	152
Missing in covariates	2926
No 2 consecutive waves	10,972
No information on state in t−1 or t0	8360
Estimation sample: Observations	21,999
Individuals	7909
Transitions per individual	
Average	2.782
Min.	1
Max.	6

Source: PASS_0614_v1 & LHG V08.01.00 – 201504

A major advantage of the PASS is that it can be linked on the individual level to administrative data from the Federal Employment Agency. Due to legal considerations, the survey information can only be linked to the administrative data of respondents who consented to linkage in the survey. We had to drop 2374 observations from our sample because the respondents did not consent to the linkage (see Table 2).

Next, the respondents consenting to linkage have to be found in the administrative data. We use the identification keys provided by the German data linkage centre to identify respondents in the administrative data. The German data linkage centre combines information on names, addresses, identification number, gender and date of birth from several administrative data sources (on dependent employment, registered unemployment and welfare benefits), which is used for record linkage to the survey data (Antoni et al. 2017; Sakshaug et al. 2017). These used administrative data sources are collected by the Federal Employment Agency and build the basis for the official statistics in Germany. Note that these data do not cover individuals who are completely out of the labour force or are not registered at the Federal Employment Agency, e.g., self-employed persons, civil servants or housewives. Therefore, not all respondents can be linked to the administrative data. We could not identify approximately 8000 observations in the administrative data and drop them from our sample.

From the remaining sample of respondents, who could be identified in the administrative data, we dropped 152 observations without information on welfare receipt in the survey. Nearly 3000 observations were dropped due to missing values in covariates. For our regression analysis, individuals must be observed for at least three times: one observation for addressing the initial condition problem (Wooldridge 2005) and two additional consecutive observations for the measurement of state dependence. This leaves us with 21,999 individual-year observations for our analysis.

The previously described matching of the survey and the administrative data may lead to a selectivity bias in our final sample and limit representativeness. The bias could arise from the exclusion of individuals, who did not give consent to linkage or who could not be identified in the data. Appendix: Table 9 provides a comparison of our final estimation sample with individuals not consenting to data linkage or not found in the administrative data. The comparison shows statistically significant differences between both groups in most socio-economic and regional characteristics. There are significantly more higher educated individuals, individuals with a migration background and individuals who live in a household with young children in the sample of unlinked observations. However, absolute differences between both groups are only small in most cases. To address the potential selectivity bias resulting from the matching procedure, we will provide the results of a robustness check based on unlinked data in Sect. 7 for our main findings.

An important methodological aspect of studying welfare benefit dynamics is the definition of the benefit receipt (Cappellari and Jenkins 2008). Königs (2014) shows that estimated transition probabilities react sensitively to whether the benefit receipt for the individual is defined at the household or individual level. Most studies use information on the benefit receipt collected at the household level, as the eligibility for most means-tested income support programmes is assessed on the income, wealth and needs of a benefit-receiving unit, i.e. the household. For the German UB II, the benefit-receiving unit generally differs from the household: The relevant benefit-receiving unit is only the (core) family (Bedarfsgemeinschaft) living in the same household, i.e., recipients with their partner and children aged below 25 years (see Sect. 2). The PASS data allow us to determine exactly the members of the benefit receiving family, in the aforementioned sense. We use this information and define the benefit receipt by the membership in a core family who receive welfare benefits in the month of the interview. Note that this definition does not allow us to exactly draw conclusions about the individual motives of under- or overreporting, because the head of the household reports benefit receipt for the household.

In PASS the question on UB II receipt is asked in the annual household interview. Households being interviewed for the first time are asked whether they received UB II at any time during the past 2 years and are also asked to indicate all spells of UB II receipt. In the interviews of wave 8 (interviews between February and September 2014) the question was:

“Now we are only interested in unemployment benefit 2 (“Arbeitslosengeld 2“), otherwise known as Hartz 4. Thinking of the time since January 2012, what about your household? Have you or has any other member of your household obtained unemployment benefit 2 at any stage since January 2012? And from when to when exactly was this the case? Please give me the month and the year. If that was the case more often than once, please begin with the first space of time.”

The answer to this question is then used to generate the information on welfare benefit receipt at the date of the interview which we use in our analysis. We do neither impute the welfare benefit receipt variable at the time of the interview nor any explanatory variable if the information is missing due to item-nonresponse. The interviews of panel households are conducted dependent on the answers regarding UB II receipt in the previous interview (roughly 1 year ago). Households that stated in the previous wave not to receive UB II are asked if they have received benefits at any time since the last interview, whereas households that stated to receive UB II are asked until when they received these benefits without interruption.

To measure under- and overreporting, we use the information on benefit receipt available in the administrative welfare records collected in the “Leistungshistorik Grundsicherung (LHG)” and the Integrated Employment Biographies (IEB) respectively. The records contain daily information on the welfare benefit receipt until 31 December 2014. In the administrative data, all members of the core family are registered as benefit recipient. While misreporting is an issue in survey data, administrative data on welfare receipt can be regarded as highly reliable since it is based on actual welfare benefit payments that local welfare offices make to welfare recipients (Eggs 2016). We define underreporting as not reporting welfare benefit receipt at the interview date in the household interview although the administrative data contains individual benefit receipt for at least 1 day in the month of the interview. Underreporting is defined for the month of the interview because the benefit is granted on a monthly basis. Correspondingly, overreporting occurs if we find no benefit receipt during the whole month of the interview in the administrative data, while in the household survey current benefit receipt was reported.

### Method

For the econometric analysis, we use a dynamic multinomial logit model with controls for observed and unobserved heterogeneity and for endogenous initial conditions. This is the most frequently applied estimation strategy in latest research on the dynamics of welfare benefit receipt. The model is based on transitions among four different labour market states, which we obtain by interacting the information on welfare benefit receipt with the employment state: welfare benefit receipt, welfare benefit receipt while having a job, inactivity and employment. For the PASS data, both information stem from the survey data. The IEB outcome variable also uses the employment state from the PASS but the welfare receipt information is taken from the IEB. Employment comprises employment subject to social security contributions as well as marginal employment.^4^ Table 6 shows the misclassification of the PASS outcome variable resulting exclusively from individuals misreporting welfare benefits in the PASS survey. The cases on the diagonal from the top left to the bottom right corner are correctly classified as they represent the cases in which the PASS information on welfare benefit receipt corresponds to the administrative information while all off-diagonal cases are falsely classified.

The probability of being in one of the four different states is based on a random utility model, which means that individual *i* chooses the labour market state *j* at the time of the interview *t* that yields the highest utility *U*. This model can be formalized as:

1 $$U_{ijt} = x^{\prime}_{it} \beta_{j} + \lambda_{j} y_{it - 1} + u_{ijt} \quad for \ i = 1, \ldots ,N; \ t = 1, \ldots ,T_{i} ; \ j = 1,2,3,4$$

where *U*_*ijt*_ depends on a vector of strictly exogenous regressors *x*_*it*_ and the corresponding state-specific coefficient vector *β*_*j*_, the labour market state in the previous period *y*_*it*−1_, the corresponding state-specific coefficient vector *λ*_*j*_ that measures state dependence and a composite error term *u*_*ijt*_. This error term can further be decomposed into $u_{ijt} = \alpha_{ij} + \varepsilon_{ijt}$, where *α*_*ij*_ represents individual and labour market state specific unobserved heterogeneity and *ɛ*_*ijt*_ is an idiosyncratic error component that is assumed to be independently distributed with a type I extreme value distribution. The inclusion of *α*_*ij*_ allows us to distinguish between true and spurious state dependence. As exogenous regressors, we include information on age, sex, education,^5^ migration background, health, partner, children, regional unemployment rate, region, and wave.

As in every dynamic panel data model allowing for the presence of unobserved heterogeneity, the problem of endogenous initial conditions arises. Transitions to the first labour market state cannot be observed because we have no information on the labour market state in the preceding period. When first observed in the data, individuals can be in a particular labour market state because they have already been in the same state in the precending period (true state dependence) or because their observed and unobserved characteristics increase the probability to be in this state (spurious state dependence). The correlation of unobserved heterogeneity with the initial benefit receipt state is responsible for the fact that the initial values of the labour market state are endogenous (Heckman 1981a).

In general, there are different ways to address the initial conditions problem. The earliest approach was proposed by Heckman (1981b). He suggested approximating the unknown density of *y*_*i*0_|*x*_*i*_, *α*_*ij*_ to remove the former conditioning on the individual specific time-invariant effect *α*_*ij*_. A simpler approach to deal with endogenous initial conditions was suggested by Wooldridge (2005). Wooldridge proposed a conditional maximum likelihood estimator by specifying a distribution of unobserved heterogeneity *α*_*ij*_ conditional on the initial observation of the welfare benefit receipt state *y*_*i*0_ and all observations of time-varying explanatory variables *x*_*i*_ that have not yet been included in *x*_*it*_ such that the model coincides with the correlated random effects model proposed by Chamberlain (1984). In empirical practice, the distribution of the unobserved individual heterogeneity is usually not specified to be conditional on all lags and leads of the time-varying explanatory variables but on individual averages of the time-varying explanatory variables $\bar{x}_{i}$ and a new random error *n*_*ij*_with $n_{ij} \left| {y_{i0} ,x_{i} } \right. \sim N\left( {0, \sigma_{n}^{2} } \right)$. $n_{ij}$ is now uncorrelated with the benefit receipt in the initial period such that the model coincides with the Mundlak (1978) quasi-fixed effects approach:

2 $$\alpha_{ij} = \gamma_{j1} y_{i0} + \bar{x}^{\prime}_{i} \gamma_{j2} + n_{ij}$$

Inserting (2) into the random utility model (1) described above yields:

3 $$U_{ijt} = x^{\prime}_{it} \beta_{j} + \lambda_{j} y_{it - 1} + \gamma_{j1} y_{i0} + \bar{x}^{\prime}_{i} \gamma_{j2} + n_{ij} + \varepsilon_{ijt}$$

The assumption that the idiosyncratic error *ɛ*_*ijt*_ follows a Type I extreme value distribution results in a dynamic multinomial logit model with random effects. The probability of individual *i* being in labour market state *j* at time *t* is thus given by:

4 $$P\left( {y_{it} = j |x_{it} ,y_{it - 1} ,\alpha_{ij} } \right) = \frac{{exp(x^{\prime}_{it} \beta_{j} + \lambda_{j} y_{it - 1} + \gamma_{j1} y_{i0} + \bar{x}^{\prime}_{i} \gamma_{j2} + n_{ij} )}}{{\mathop \sum \nolimits_{h = 1}^{4} exp(x^{\prime}_{it} \beta_{h} + \lambda_{h} y_{it - 1} + \gamma_{h1} y_{i0} + \bar{x}^{\prime}_{i} \gamma_{h2} + n_{ih} )}}$$

Setting the coefficients $\beta_{4} , \lambda_{4} , \gamma_{41} ,\gamma_{42}$ and the unobserved heterogeneity *n*_*i*4_ to zero for the labour market state “employment” (*j *= 4), we are able to estimate a dynamic multinomial logit model with random effects. Due to the non-linearity of the model, the size of the estimated coefficients for the lagged labour market state dummies (and all other coefficients) cannot be interpreted directly, and thus we calculate the marginal effects of all variables on the four different response probabilities.

The empirical model yields credible results if the following identifying assumptions are met. First, the model assumes that state dependence follows a first-order Markov process, meaning that only the labour market state of the last period is correlated with the labour market state of the current period. Second, the model assumes that after controlling for initial labour market states and the averages of time-varying observable characteristics any remaining unobserved heterogeneity is uncorrelated with the regressors (including the lagged labour market state).

To assess whether measurement error induced by the misreporting of welfare benefits affects the estimate of true state dependence, we run two separate dynamic multinomial logit regressions and compare the obtained marginal effects. The only difference between the two regressions is that the information on whether a person receives welfare benefits stems from different sources: In the first regression, the information is taken from the PASS panel survey, and in the second regression, we take the information from the administrative records of the German Federal Employment Agency. However, in both regressions we use information on the employment state from the PASS panel survey to construct the four categories of our outcome variable. By comparing the two different estimates of structural state dependence, we can assess whether using survey data yields correct estimates or whether misreported welfare benefits endanger the validity of the results.

## Descriptive analysis

### Extent and nature of misreporting

According to administrative records, we have 7102 person-year observations (3211 individuals) in which employed and non-employed person received welfare benefits in the month of the interview (Table 3). For 6489 observations, this agrees with the information in the survey, and in 613 cases, respondents did not report the benefit receipt in the PASS survey. This yields an underreporting rate of 8.6% (13.9% weighted). Regarding all non-recipients, the share of respondents who overreported welfare benefit receipt in our sample amounted to 1.6% (0.3% weighted) or 238 observations.

**Table 3:** Extent of benefit misreporting

Estimation sample (N)	21,999
…thereof with benefit receipt (administrative data, N)	7102
…thereof without reported benefit receipt in the survey data (underreporting, N)	613
…thereof with underreporting in only one wave (N)	428
…thereof with underreporting in two waves (N)	112
…thereof with underreporting in more than two waves (N)	73
*Underreporting rate*	*0.086*
…thereof without benefit receipt (administrative data, N)	14,897
…thereof with reported benefit receipt in the survey data (overreporting, N)	238
*Overreporting rate*	*0.016*

Source: PASS_0614_v1 & LHG V08.01.00 – 201504

From Table 3 we see that 428 respondents underreport welfare benefit receipt only once which makes about two-third of all underreporting cases (428/613). Approximately half of them have received welfare benefits only in this wave according to the administrative data. The remaining third of underreporting cases consists of 56 respondents (112 observations) who underreported twice and 21 respondents (73 observations) who underreported in more than two waves. Thus, we find no clear pattern in the way that the majority of under-reporters tend to underreport permanently or occasionally. In 44% of the underreporting cases, the respondents underreported benefit receipt in each wave in which they received benefits, according to administrative records (permanent underreporting). However, most of these cases are the respondents who were observed only once as recipients.

Table 4 shows how the share of under-reporters changes depending on the number of waves in which respondents are observed in our analysis sample and in which they have received welfare benefits, according to administrative records. For panel participation, we see a slight decrease in underreporting from 10.6% for the subgroup who participated in three waves to 8.3% for those who participated in all 8 waves. This finding is in line with Eggs (2016), who shows that the accuracy of survey responses concerning benefit receipt increases from wave to wave for the PASS. According to his findings, underreporting was especially high in the first two PASS waves. However, since it is necessary to observe individuals for at least two consecutive waves (t1, t2) and in one previous wave (t0) to measure transitions and the initial condition, we do not have individuals who participated only in the first two waves in our analysis sample.

**Table 4:** Benefit underreporting by panel participation and benefit receipt in the analysis sample

	Participation in … waves		Benefit receipt in … wave(s)	
	Underreporting rate	Observations	Underreporting rate	Observations
1			0.140	1350
2			0.090	1794
3	0.106	649	0.078	1092
4	0.088	1250	0.063	1124
5	0.098	716	0.051	860
6	0.087	1015	0.070	882
7	0.075	1400		
8	0.083	2072		
All	0.086	7102	0.086	7102

Source: PASS_0614_v1 & LHG V08.01.00 – 201504

For benefit receipt, the results point to a stronger relationship in which underreporting seems to be higher for short-term recipients, i.e., those in our analysis sample who received benefits in only one or two waves. The share of under-reporters included in the sample declines from 14.0% for the group who received benefits in only one wave to approximately 5.1% for respondents with benefit receipt in 5 waves.

Taken together, the figures on the length of benefit receipt and underreporting do not allow any clear conclusions to be drawn on the impact of underreporting benefit receipt on the transitions measured in the survey data. On the one hand, those who received benefits only in one wave and were classified as under-reporters have never been observed as benefit recipients in the survey data, which would lead to an underestimation of transitions into and out of benefit receipt in the survey data. On the other hand, the observed dynamics in welfare benefit receipt could be overestimated relying on survey data because most respondents underreport only once but receive benefits for more than one wave. This should result in artificial transitions into and out of the welfare system. Since the observed underreporting rate is small and no clear dynamic pattern of underreporting emerges, the effects on observed transitions should be minor.

### Characteristics of under-reporters

Table 5 depicts the marginal effects of a regression of underreporting on respondents' characteristics for welfare benefit recipients who reported and those who did not report benefit receipt in our analysis sample.

**Table 5:** Regression of underreporting on socio-demographic and household characteristics: marginal effects

	Marg. effect	Std. error
Age in years	− 0.0019***	0.0004
Male	0.0036	0.0081
Education (Casmin, categories 1–3)	0.0187**	0.0059
Health restrictions	− 0.0632***	0.0084
Migration background	0.0066	0.0092
Partner	0.0273**	0.0080
Child < 2 years	− 0.0525*	0.0189
Child 2 to 3 years	− 0.0458*	0.0157
Child 4 to 6 years	− 0.0006	0.0110
Child 7 to 16 years	− 0.0256**	0.0086
Living in East Germany	− 0.0109	0.0098
Local unemployment rate (annual average)	− 0.0031*	0.0015

Marginal effects on the probability to underreport UB II benefits based on the sample of 7102 respondents who could be identified as recipients in the administrative data (613 Underreporters, 6489 respondents who reported correctly). Standard errors clustered at the individual level. ***/**/* denote statistical significance at the 0.001, 0.01, 0.05 levels

Source: PASS_0614_v1 & LHG V08.01.00 – 201504

The results show that higher educated respondents, respondents without health restrictions and who live together with a partner in a household without young children in the household have a statistically significant higher probability to not correctly report benefit receipt in the survey. These figures indicate that benefit recipients with relatively good labour market prospects tend to underreport welfare benefits more often, which is completely in line with the results of Bruckmeier et al. (2014). A possible explanation for this result could be that the recipients do not identify themselves with benefit recipients because the amount of benefits is low or they expect to receive benefits only temporarily. It could also be argued that if the benefit is prone to be seen as a stigma, the stigma costs are higher for those with good labour market prospects, as benefit dependence for them is less accepted within society.

### Misreporting and transitions between employment and welfare receipt

The finding that underreporting seems to correlate with certain characteristics is of particular interest for our analysis, as we do not only distinguish between benefit receipt and non-receipt. In addition to benefit receipt, we account for employment when defining the four outcome states: benefit receipt only, employed with benefit receipt, inactive without benefit receipt and employed without benefit receipt. Table 6 shows the effects of under- and overreporting on our four labour market categories. Due to the underreporting of welfare receipt, 193 cases are falsely classified as inactive without benefit receipt and 420 cases are falsely classified as employed without benefit receipt when using PASS survey data. Considering that the group of employed benefit recipients is only half the size of the non-employed benefit recipients, these figures show that the state “benefit receipt and employment” is more strongly affected by underreporting. This is in line with the expectations arising from the results on the composition of the under-reporters. Most over-reporters, in contrast, would be correctly classified as inactive without employment (135 observations). The incidence of overreporting benefit receipt is much more pronounced among the inactive compared to the employed. The share of over-reporters among the inactive amounts to 8.5% (135/1580) while among the employed the share is about 0.7% (103/13,317). Since non-employed individuals who receive other kinds of benefits (e.g. unemployment benefit I) are included in the inactivity group we assume that the major driver for overreporting is confusion about different benefits.

**Table 6:** Classification of outcomes using survey data only and correcting for overreporting and underreporting

Outcomes survey data (PASS)	Outcomes corrected survey data (IEB)				Total
	Benefit receipt	Benefit receipt and employment	Inactivity	Employment	
Benefit receipt	4215	–	135	–	4350
Benefit receipt and employment	–	2274	–	103	2377
Inactivity	193	–	1445	–	1638
Employment	–	420	–	13,214	13,634
Total	4408	2694	1580	13,317	21,999

Source: PASS_0614_v1 & LHG V08.01.00 – 201504

Table 7 summarizes all transitions between the time of the previous interview t−1 and the time of the current interview t in the two different data sources for the four states. Panel A depicts the welfare transitions when using PASS survey data, and Panel B depicts welfare transitions that are observed for the same individuals when both benefit under- and overreporting are corrected based on the administrative records. First, Table 7 shows that the effects of the correction on the transition rates are rather small. Thus, we find no difference in the proportion of recipients who also receive benefits in the following wave (74%). With regard to exits from benefit receipt, the table shows that the incidence of benefit misreporting leads to more exits in survey data. 5% of the welfare benefit recipients exit benefit receipt into inactivity and 9% into employment compared to 4% and 7% in the administrative data. Using the misreporting corrected measure from administrative data, we can see that a larger proportion of benefit recipients is still dependent on benefits after finding a job (14% vs. 12%). Consistent with this finding, slightly more employed recipients remain in the status “benefit receipt and employment” in the next wave when the administrative data is considered (61% vs. 60%). We also find that inflows into the welfare system in the survey data are somewhat higher for groups of inactive respondents (13% vs. 10%), whereas in the administrative data, more inactive respondents remain in this state in the next year (57% vs. 52%). This finding can be explained by two distinct mechanisms. Both benefit recipients who underreport in t−1 but not in t and inactive individuals in t−1 who overreport in t lead to higher inflows into benefit receipt and lower persistence in inactivity when using survey data.

**Table 7:** Transitions among the four labour market states between t−1 and t

State at time t−1	State at time t			
	Benefit receipt	Benefit receipt and employment	Inactivity	Employment
*Panel A: PASS survey data*				
Benefit receipt	3480	572	239	404
	74%	12%	5%	9%
Benefit receipt and employment	468	1492	54	480
	19%	60%	2%	19%
Inactivity	218	49	858	522
	13%	3%	52%	32%
Employment	184	264	487	12,228
	1%	2%	4%	93%
Total	4350	2377	1638	13,634
*Panel B: administrative data*				
Benefit receipt	3567	688	196	339
	74%	14%	4%	7%
Benefit receipt and employment	546	1755	41	527
	19%	61%	1%	18%
Inactivity	151	39	881	481
	10%	3%	57%	31%
Employment	144	212	462	11,970
	1%	2%	4%	94%
Total	4408	2694	1580	13,317

First row shows the absolute numbers of labour market transitions between t−1 and t. Second row shows labour market transitions between t−1 and t as a share of the labour market state in t−1

Source: PASS_0614_v1 & LHG V08.01.00 – 201504

In summary, the description of the transitions shows that the impact of misreporting on observed transitions is rather small. Furthermore, misreporting seems to lead to slightly higher observed dynamics in the survey data concerning both inflows into and outflows from the welfare system. In addition, the transitions of employed beneficiaries are more affected since, proportionally, underreporting is observed more frequently among this group.

## Estimation results

The average partial effects (APE) of the different states in the preceding period on the four different response probabilities based on the unweighted estimates are shown in Table 8.^6^ Panel A depicts the results for the self-indicated welfare measures obtained from the PASS survey data, and Panel B corresponds to the misreporting-corrected measures from the administrative records. Overall, we can see that the differences between the two different measures are rather small: All significant average partial effects go in the same direction and remain significant when underreporting and overreporting are corrected based on administrative records (except for the effect of welfare receipt in t−1 on inactivity which becomes insignificant). In addition, in absolute numbers, the effects do not differ considerably between the data sources.^7^

**Table 8:** Average partial effects of lagged labour market state on the probability to be in a given state in t

	Welfare receipt	Welfare and employment	Inactivity	Employment
*Panel A: PASS survey data*				
Welfare receipt in t−1	0.4228***	0.0884***	0.0240**	− 0.535***
	[0.3867; 0.4590]	[0.0696; 0.1073]	[0.0058; 0.0422]	[− 0.5757; − 0.4948]
Welfare and Employment in t−1	0.1192***	0.3484***	− 0.0237***	− 0.444***
	[0.0985; 0.1400]	[0.3130; 0.3838]	[− 0.0377; − 0.0098]	[− 0.4818; − 0.4059]
Inactivity in t−1	0.1162***	0.0022	0.2752***	− 0.394***
	[0.0925; 0.1400]	[− 0.0127; 0.0171]	[0.2351; 0.3152]	[− 0.4345; − 0.3527]
*Panel B: administrative data*				
Welfare receipt in t−1	0.4543***	0.1189***	0.0077	− 0.581***
	[0.4149; 0.4938]	[0.0976; 0.1402]	[− 0.0104; 0.0258]	[− 0.6242; − 0.5378]
Welfare and Employment in t−1	0.1307***	0.3550***	− 0.0361***	− 0.450***
	[0.1100; 0.1513]	[0.3193; 0.3907]	[− 0.0490; − 0.0231]	[− 0.4890; − 0.4102]
Inactivity in t−1	0.1021***	0.0070	0.3087***	− 0.418***
	[0.0774; 0.1268]	[− 0.0092; 0.0231]	[0.2663; 0.3511]	[− 0.4604; − 0.3752]

Average partial effects based on coefficients shown in Appendix: Tables 10 and 11 are depicted in the first rows. Second rows show the 95% confidence interval in brackets using clustered standard errors at the individual level. Base category: Employment in t−1. ***/**/* denote statistical significance at the 0.001, 0.01, 0.05 levels, respectively. Average partial effects of other explanatory variables can be found in Appendix: Tables 13 and 14

Source: PASS_0614_v1 & LHG V08.01.00 – 201504

### APE of receiving welfare benefits in t−1

Receiving welfare benefits in t−1 compared to being employed in t−1 is, on average, associated with an increase of the probability to receive welfare benefits in t by 42.28 percentage points when using survey data, but the probability increases to about 45.43 percentage points when using the corrected measure. The lower state dependence in the survey data can be explained by two different facts.

First, due to the incidence of misreporting the welfare benefits, the composition of the base outcome between the two data sources differs. When using administrative records, the base category solely consists of employed non-recipients in t−1, but the base category in the estimations with the survey data, in addition to employed individuals, includes underreporting employed welfare benefit recipients in t−1.

Since employed welfare recipients tend to move more often into welfare than employed non-recipients, state dependence in welfare benefit receipt is underestimated when survey data is being used. Second, when individuals receive welfare benefits in t−1 and t but only indicate that they receive benefits in t−1, they only contribute to the state dependence estimate when using administrative data, while they are coded as inactive in t when using survey data. The average partial effects for welfare benefit receipt on the inactivity response probability (column 3 of Table 8) indicates that underreporting of welfare receipt is, indeed, an important factor. Compared to being employed in t−1, receiving welfare benefits in t−1 is associated with a higher chance to move into the inactivity category in t. The likelihood increases by approximately 2.4 percentage points when using survey data but only by approximately 0.8 percentage points (not significant) when using the corrected measure from administrative records.

The average partial effect of receiving welfare benefits in t−1 on the probability of being in the welfare and employment category in t is approximately 3 percentage points lower when survey data are used than when administrative data are used (11.9 vs 8.9 percentage points). Again, the difference emerges from the different base categories between the different data sources. Since employed welfare recipients display a higher probability of being employed welfare recipients in t than do employed non-recipients, the average partial effect of receiving welfare benefits is lower when comparing to both employed welfare recipients and employed non-recipients as opposed to employed non-recipients only.

Relative to being employed in t−1, receiving welfare benefits in t−1 is associated with a 4.6-percentage points lower likelihood (− 53.5 vs. − 58.1 percentage points) of being employed when using the survey welfare measure than when administrative data are used. This finding can be explained by two factors. First, the base category in survey data consists of both employed non-recipients and employed recipients who do not report benefit receipt, but it consists solely of employed non-recipients when using administrative data. Since the probability to exit from benefit receipt into employment for employed welfare benefit recipients is usually lower than the probability of employed non-recipients to stay in employment, the average partial effect of welfare benefit receipt in t−1 on the probability to be employed is lower when using survey data. Second, some welfare benefit recipients may not continue to report welfare benefits after having found a job, as they do not perceive themselves as benefit recipients because they are regularly employed or may not know that their household still receives benefits due to the low amount of benefits they receive.

### APE of welfare benefit receipt and employment in t−1

Looking at the average partial effect of being in the welfare and employment category in t−1, we see similar but less pronounced effects compared to receiving welfare in t−1. The average partial effects of being in the welfare and employment category compared to those of receiving welfare benefits only indicate that having a job while receiving welfare benefits, on average, is associated with better chances to exit welfare benefit receipt and to move into the employment category. Generally, it can be stated that employed welfare benefit recipients lie between non-employed welfare recipients and employed non-recipients, as already turned out in the descriptive analysis. When using survey data to measure state dependence in the welfare and employment category, the transition probability into welfare benefit receipt in t is underestimated by less than 1 percentage point. Thus, though using survey data with a fair amount of welfare benefit misreporting by respondents, the average partial effects of past benefit receipt are not biased substantially.

### APE of being inactive in t−1

Being inactive in t−1 compared to being employed is, on average, associated with a one percentage points higher probability to move into welfare benefit receipt (11.6 vs. 10.2 percentage points), when using the self-reported welfare measure from the PASS survey data. State dependence in inactivity is estimated to be approximately 3 percentage points smaller (27.5 vs 30.9 percentage points), and the probability to exit from inactivity into employment is approximately 2 percentage points (39.4 vs 41.8 percentage points) lower when using the PASS survey data. The major explanation for this finding is that welfare benefit recipients without employment are coded as inactive when they do not report that their household receives benefits. Since some of these underreporting welfare benefit recipients state in the next period that they do receive benefits, we see a slightly higher probability to move into welfare benefit receipt and a lower probability to stay in inactivity.

### Other findings

Comparing our estimated state dependence to the ones of Wunder and Riphahn (2014) and Königs (2014), we see a distinctly higher estimated state dependence in welfare benefit receipt in our results. This might be explained by the specific sample design of the PASS which might lead to a higher number of long-term benefit recipients compared to the SOEP. The additional split of welfare recipients into employed and non-employed welfare recipients may also contribute to this finding. Since welfare recipients employed in t−1 are less likely to receive benefits in t than non-employed recipients, the estimated state-dependence in benefit receipt should be lower if all welfare recipients—employed or not—are measured in one category as in Königs (2014) and Wunder and Riphahn (2014). Due to the complex survey design, using sampling weights should be appropriate to receive representative results that are comparable to other studies. However, in our case a weighted estimation was not possible, because the estimates did not converge in the case of an outcome with four different categories. As we are not primarily interested in determining the absolute level of state dependence in German welfare benefit receipt, but in determining the role of misreporting for the estimated state dependence, we do not use sampling weights. Instead, we include a dummy variable “population sample” in the main estimations, which should capture the sampling design. Furthermore, we run an estimation on the bivariate outcome “welfare receipt” as an additional check displayed in Appendix: Table 12. We run these estimations weighted, unweighted for the full sample and the population sample only and unweighted but including the population sample dummy.

Just like for the main estimations studying four labour market states as outcomes, the unweighted estimates of state dependence in welfare receipt are similar for the two data sources (56% vs 58%). The state dependence is reduced after including the population sample dummy (52% vs 56%) and further reduced when estimated for the population sample only (38% vs 42%) or using sampling weights (39% vs 42%) or. Differences between the two data sources in the estimated state dependence based on PASS and based on the IEB stay small, but the estimated state dependence is still considerably stronger when using the population sample only or in weighted regression than estimates based on SOEP data. Possible explanations for the different results compared to studies using the SOEP lie in the different sampling and the different survey methods concerning the information on UB II receipt. Different to the SOEP, PASS uses dependent interviewing. Explaining the different results in more detail would be an interesting issue for future research.

Concerning the effects of the control variables, Appendix: Tables 13 and 14 show the average partial effects of the initial conditions and the other explanatory variables. Controlling for initial conditions was necessary, as seen by the significant average partial effects of some states in t0. Again, the estimated average partial effects do not differ significantly between the different welfare measures. Generally, it can be seen that the average partial effects of the initial states go in the same direction as those of the preceding state but are less pronounced.

The other explanatory variables all show the expected signs on the four different response probabilities. Individuals belonging to the population sample of the PASS data have a higher chance to be employed and a lower chance of being in one of the two welfare categories. Higher education is associated with a lower risk of being in one of the two welfare categories. Individuals with health restrictions and with a small child below the age of 2 show a higher probability of receiving welfare benefit. Health restrictions are associated with a lower chance of employment, as seen by the negative average partial effects on the probability of being in the welfare and employment or employment category. Generally, it can be stated that the effects do not greatly differ between the different welfare measures.

Altogether, the results indicate that state dependence in welfare benefit receipt is only slightly underestimated when self-reported welfare measures from survey data are being used. The same holds for state dependence in the other categories. The average partial effects of past benefit receipt from survey data do not differ considerably with the misreporting corrected welfare measures from the administrative records. Thus, we argue that the use of survey data with a similar amount of misreporting respondents does not severely bias the estimates of state dependence.

## Conclusion

In this study, we analyse the impact of benefit misreporting in survey data on the estimated state dependence in benefit receipt. Our results make a relevant contribution to the literature on state dependence in welfare receipt, which is mostly based on survey data. Our data allow us to determine the mismeasurement of benefit receipts directly by linking survey data with high quality administrative data. In survey data, we find that in 8.6% of the cases with benefit receipt individuals do not report that they receive benefits (underreporting) and in 1.6% of the cases without benefit receipt individuals report that they receive welfare benefits (overreporting). The descriptive analysis shows that the impact of misreporting on observed transitions between benefit receipt and non-receipt is rather small. Misreporting leads to slightly higher dynamics in the survey data concerning inflows into and outflows from the welfare system. This is explained by the fact that most respondents underreport welfare benefit receipt for only one period but receive benefits for more than one period. This leads to falsely measured inflows and outflows. In addition, transitions of the employed recipients are more affected by misreporting than the transitions of not employed recipients. This is the case because employed welfare recipients with relatively good labour market prospects tend to underreport welfare benefits more frequently.

To assess the effects of misreporting on the estimates of state dependence, we estimate two commonly used dynamic random effects models and compare the results. One model is based on the original survey data whereas the other uses the survey data where the information on welfare receipt is corrected for misreporting based on the administrative records. Altogether, the results indicate that state dependence in welfare benefits receipt is only slightly underestimated when the self-reported welfare measures from the survey data are being used. The same holds for true state dependence in the other studied labour market states. Thus, we argue that the use of survey data with a similar amount of misreporting respondents does not severely bias the estimates of state dependence in welfare receipt.

## Appendix

See Tables 9, 10, 11, 12, 13, 14, 15.

**Table 9:** Individual and household characteristics across the linked and unlinked sample (means)

	Linked sample (N = 21,999)	Unlinked sample (N = 10,099)
Age in years	43.63	42.93***
Male	0.43	0.47***
Low education	0.33	0.33
Intermediate education	0.49	0.43***
High education	0.17	0.24***
Health restrictions	0.27	0.24***
Migration background	0.20	0.24***
Partner	0.60	0.65***
Child < 2 years	0.04	0.06***
Child 2 to 3 years	0.07	0.08
Child 4 to 6 years	0.12	0.12
Child 7 to 16 years	0.35	0.32**
Local unemployment rate (annual average)	7.80	7.26***
Living in East Germany	0.27	0.22***
Subsample 2	0.44	0.47*

The linked sample describes our final sample for the analysis and estimation (N = 21,999). The unlinked sample consists of individuals without consent to data linkage or who were not identified in the administrative data. To make the sample comparable to our estimation sample, observations from wave 1, pensioners and young people (< 25 years), pupils, students, civil servants, individuals incapable of working and self-employed were removed from the sample. Asterisks ***/**/* denote significantly different means between both groups at the significance level of 0.001/0.01/0.05 levels

Source: Source: PASS_0614_v1

**Table 10:** Multinomial logit regression results (PASS)—coefficients

	Benefit receipt		Benefit receipt and employment		Inactivity	
	Coeff.	S.E.	Coeff.	S.E.	Coeff.	S.E.
Benefit receipt in t−1	5.0089***	0.1222	3.2356***	0.1136	2.0559***	0.1204
Benefit receipt and employment in t−1	3.2084***	0.1231	3.9670***	0.1107	0.6272***	0.1721
Inactivity in t−1	2.7759***	0.1391	1.1506***	0.1821	2.9883***	0.1123
Benefit receipt in t0	1.7534***	0.1378	0.9284***	0.1280	1.0638***	0.1258
Benefit receipt and employment in t0	0.8209***	0.1412	1.0580***	0.1317	0.2962	0.1559
Inactivity in t0	1.2149***	0.1435	0.4815**	0.1512	1.3501***	0.1130
Population sample	− 0.9526***	0.1080	− 1.0778***	0.1167	− 0.2895**	0.0931
Age (in years)	0.0152***	0.0046	0.0290***	0.0047	0.0024	0.0050
Male	0.0317	0.0750	− 0.2323**	0.0764	− 0.6482***	0.0782
Intermediate education	− 0.6340***	0.0791	− 0.4815***	0.0811	− 0.3014***	0.0837
High education	− 1.0814***	0.1219	− 0.8814***	0.1227	− 0.5040***	0.1106
Health restriction	0.5832***	0.1317	0.0201	0.1392	0.3749**	0.1417
Migration Background	0.1588	0.0883	0.2329**	0.0860	0.2095*	0.0893
Partner in same HH	− 1.0662***	0.2368	− 0.6654**	0.2489	− 0.1677	0.2622
Child < 2 years old	1.2890***	0.2118	0.7480**	0.2428	1.4878***	0.1845
Child 2–3 years old	0.4752*	0.2032	0.7053**	0.2154	0.2797	0.1689
Child 4–6 years old	0.0342	0.1842	0.5608**	0.1831	− 0.0322	0.1547
Child 7–16 years old	0.3058	0.1739	0.5866***	0.1714	− 0.1857	0.1586
Regional unemployment rate	0.0940**	0.0311	0.0928**	0.0308	0.0582	0.0322
Eastern Germany	0.3981	1.0921	0.3123	0.7108	− 1.0114	0.8594
M: Health restriction	0.7814***	0.1693	0.4949**	0.1750	0.3968*	0.1809
M: Partner in same HH	0.1436	0.2522	− 0.0556	0.2628	0.3880	0.2782
M: Child < 2 years old	0.3083	0.3795	0.0397	0.4074	0.2322	0.3552
M: Child 2–3 years old	0.1600	0.3282	− 0.0200	0.3400	0.0412	0.2910
M: Child 4–6 years old	0.1265	0.2539	− 0.4625	0.2544	0.0538	0.2294
M: Child 7–16 years old	− 0.1758	0.1942	− 0.1777	0.1936	0.0678	0.1824
M: Regional unemployment rate	0.0150	0.0301	0.0044	0.0300	− 0.0115	0.0315
M: Eastern Germany	− 0.4857	1.0931	− 0.3727	0.7126	0.7945	0.8604
Wave 4	0.4489***	0.1300	0.5118***	0.1344	− 0.1953	0.1223
Wave 5	0.0954	0.1222	0.0446	0.1261	− 0.3006*	0.1183
Wave 6	0.1244	0.1249	0.1017	0.1304	− 0.3620**	0.1216
Wave 7	0.2867*	0.1198	0.2216	0.1228	− 0.1799	0.1169
Wave 8	0.3371**	0.1225	0.3311**	0.1270	− 0.2541*	0.1198
RE1/RE2/RE3	1.0000		1.0000		1.0000	
Constant	− 5.9643***	0.2915	− 5.5895***	0.2972	− 3.8094***	0.2826
Var(RE1)/Var(RE2)/Var(RE3)	0.8546***	0.2091	0.5998***	0.1647	0.7568***	0.1748

Base category: employment. N = 21,999; M = individual average of covariate, S.E.: Standard errors clustered at the individual level. ***/**/* denote statistical significance at the 0.001, 0.01, 0.05 levels, respectively

Source: PASS_0614_v1

**Table 11:** Multinomial logit regression results (administrative data)—coefficients

	Benefit receipt		Benefit receipt and employment		Inactivity	
	Coeff.	S.E.	Coeff.	S.E.	Coeff.	S.E.
Benefit receipt in t−1	5.3885***	0.1334	3.7550***	0.1206	1.9462***	0.1279
Benefit receipt and employment in t−1	3.3910***	0.1287	4.1235***	0.1173	0.1580	0.1933
Inactivity in t−1	2.7994***	0.1525	1.3004***	0.2003	3.1399***	0.1132
Benefit receipt in t0	1.8975***	0.1653	1.2272***	0.1496	1.2336***	0.1422
Benefit receipt and employment in t0	1.0210***	0.1635	1.3828***	0.1533	0.4508**	0.1627
Inactivity in t0	0.9596***	0.1716	0.3729*	0.1807	1.3818***	0.1203
Population sample	− 0.7084***	0.1091	− 0.8628***	0.1206	− 0.2343*	0.1011
Age (in years)	0.0138**	0.0047	0.0268***	0.0049	0.0067	0.0052
Male	0.0564	0.0766	− 0.1551*	0.0785	− 0.6392***	0.0805
Intermediate education	− 0.5762***	0.0805	− 0.3475***	0.0816	− 0.2148*	0.0869
High education	− 1.0006***	0.1275	− 0.7386***	0.1235	− 0.4383***	0.1147
Health restriction	0.6462***	0.1337	0.0817	0.1335	0.3410*	0.1444
Migration Background	0.1022	0.0897	0.2026*	0.0865	0.2484**	0.0926
Partner in same HH	− 1.3156***	0.2495	− 0.9690***	0.2602	− 0.0407	0.2841
Child < 2 years old	1.1945***	0.2248	0.4638	0.2622	1.4554***	0.1915
Child 2–3 years old	0.3353	0.1998	0.3674	0.2203	0.1995	0.1771
Child 4–6 years old	0.0623	0.1808	0.4023*	0.1891	− 0.1650	0.1650
Child 7–16 years old	0.2208	0.1881	0.4123*	0.1867	− 0.2075	0.1652
Regional unemployment rate	0.0794*	0.0312	0.0787*	0.0322	0.0783*	0.0331
Eastern Germany	0.5492	1.1227	1.0949	0.8585	− 0.7338	0.8773
M: Health restriction	0.5887***	0.1703	0.3056	0.1718	0.5467**	0.1825
M: Partner in same HH	0.4448	0.2651	0.2836	0.2747	0.2622	0.3009
M: Child < 2 years old	0.1596	0.4039	0.1642	0.4377	0.4151	0.3719
M: Child 2–3 years old	0.1421	0.3385	− 0.0007	0.3534	0.0857	0.3091
M: Child 4–6 years old	0.2076	0.2538	− 0.1685	0.2605	0.1422	0.2415
M: Child 7–16 years old	− 0.1352	0.2078	− 0.1082	0.2071	0.0338	0.1912
M: Regional unemployment rate	0.0205	0.0301	0.0009	0.0313	− 0.0367	0.0324
M: Eastern Germany	− 0.6178	1.1229	− 1.1527	0.8605	0.4738	0.8797
Wave 4	0.2708*	0.1306	0.3686**	0.1311	− 0.0677	0.1275
Wave 5	− 0.1063	0.1263	− 0.1123	0.1269	− 0.1505	0.1241
Wave 6	0.0782	0.1288	0.1214	0.1315	− 0.2549*	0.1292
Wave 7	0.1968	0.1230	0.1756	0.1258	− 0.0631	0.1230
Wave 8	0.1144	0.1263	0.0979	0.1302	− 0.1333	0.1254
RE1/RE2/RE3	1.0000		1.0000		1.0000	
Constant	− 6.0222***	0.3056	− 5.7467***	0.3081	− 4.2602***	0.3011
Var(RE1)/Var(RE2)/Var(RE3)	0.7310***	0.2179	0.6618***	0.1799	0.7087***	0.1796

Base category: employment. N = 21,999; M = individual average of covariate, S.E.: Standard errors clustered at the individual level. ***/**/* denote statistical significance at the 0.001, 0.01, 0.05 levels, respectively

Source: PASS_0614_v1 & LHG V08,01,00 – 201504

**Table 12:** Average partial effects on the probability to receive welfare benefits in t (bivariate results)

	PASS				IEB			
	(1)	(2)	(3)	(4)	(1)	(2)	(3)	(4)
	APE	APE	APE	APE	APE	APE	APE	APE
Benefit receipt in t−1	0.563*** (0.017)	0.521*** (0.017)	0.381*** (0.058)	0.389*** (0.033)	0.582*** (0.018)	0.556*** (0.018)	0.421*** (0.066)	0.419*** (0.034)
Benefit receipt in t0	0.095*** (0.008)	0.068*** (0.007)	0.066*** (0.016)	0.060*** (0.009)	0.115*** (0.010)	0.087*** (0.009)	0.056*** (0.016)	0.054*** (0.007)
Age (in years)	0.001*** (0.000)	0.001*** (0.000)	0.000 (0.000)	0.000 (0.000)	0.001** (0.000)	0.001*** (0.000)	0.000 (0.000)	0.000 (0.000)
Male	0.003 (0.004)	0.003 (0.004)	− 0.003 (0.003)	0.000 (0.003)	0.006 (0.004)	0.006 (0.004)	0.000 (0.003)	0.000 (0.003)
Intermediate education	− 0.040*** (0.005)	− 0.039*** (0.005)	− 0.017*** (0.004)	− 0.015*** (0.003)	− 0.031*** (0.004)	− 0.031*** (0.004)	− 0.014*** (0.004)	− 0.008* (0.003)
High education	− 0.069*** (0.007)	− 0.066*** (0.007)	− 0.025*** (0.005)	− 0.029*** (0.004)	− 0.054*** (0.007)	− 0.053*** (0.007)	− 0.023*** (0.005)	− 0.023*** (0.004)
Health restriction	0.016* (0.008)	0.017* (0.008)	0.006 (0.006)	0.007 (0.006)	0.017* (0.007)	0.018* (0.007)	0.005 (0.006)	0.005 (0.005)
Migration Background	0.014** (0.005)	0.010* (0.005)	0.007 (0.004)	0.011** (0.004)	0.008 (0.005)	0.006 (0.005)	0.005 (0.004)	0.008* (0.004)
Partner in same HH	− 0.058*** (0.015)	− 0.058*** (0.015)	− 0.013 (0.010)	− 0.022* (0.009)	− 0.071*** (0.016)	− 0.071*** (0.015)	− 0.022 (0.013)	− 0.040** (0.015)
Child < 2 years old	0.040** (0.013)	0.037** (0.013)	0.006 (0.011)	0.034* (0.016)	0.030* (0.012)	0.028* (0.012)	0.004 (0.011)	0.026 (0.015)
Child 2–3 years old	0.038** (0.012)	0.039** (0.012)	0.027* (0.012)	0.023* (0.011)	0.025* (0.011)	0.025* (0.011)	0.029* (0.014)	0.010 (0.008)
Child 4–6 years old	0.021* (0.010)	0.019 (0.010)	0.004 (0.008)	0.020 (0.012)	0.020* (0.009)	0.018 (0.010)	0.013 (0.010)	0.007 (0.006)
Child 7–16 years old	0.034*** (0.009)	0.034*** (0.009)	− 0.002 (0.007)	0.007 (0.007)	0.024* (0.010)	0.024* (0.010)	0.013 (0.008)	− 0.003 (0.007)
Regional unemployment rate	0.006*** (0.002)	0.006** (0.002)	0.001 (0.002)	0.000 (0.001)	0.004* (0.002)	0.004* (0.002)	0.000 (0.002)	0.000 (0.001)
Eastern Germany	0.051 (0.055)	0.051 (0.055)	− 0.016 (0.009)	0.010 (0.015)	0.059 (0.054)	0.058 (0.053)	− 0.016 (0.010)	0.001 (0.019)
M: Health restriction	0.050*** (0.009)	0.048*** (0.009)	0.017* (0.007)	0.012* (0.006)	0.036*** (0.009)	0.034*** (0.009)	0.015* (0.007)	0.011* (0.005)
M: Partner in same HH	− 0.013 (0.014)	− 0.005 (0.014)	− 0.008 (0.009)	− 0.005 (0.007)	0.008 (0.013)	0.012 (0.014)	− 0.003 (0.010)	0.003 (0.008)
M: Child < 2 years old	0.006 (0.022)	0.010 (0.022)	0.005 (0.017)	− 0.027 (0.015)	− 0.003 (0.022)	0.002 (0.022)	− 0.004 (0.019)	− 0.011 (0.014)
M: Child 2–3 years old	0.010 (0.019)	0.007 (0.019)	− 0.025 (0.013)	− 0.009 (0.014)	0.003 (0.018)	0.003 (0.018)	− 0.021 (0.015)	− 0.011 (0.015)
M: Child 4–6 years old	− 0.014 (0.014)	− 0.009 (0.014)	0.013 (0.010)	− 0.007 (0.008)	− 0.004 (0.013)	0.000 (0.013)	0.007 (0.011)	0.017 (0.011)
M: Child 7–16 years old	− 0.017 (0.010)	− 0.016 (0.010)	− 0.002 (0.008)	− 0.005 (0.007)	− 0.012 (0.010)	− 0.011 (0.011)	− 0.011 (0.007)	0.009 (0.008)
M: Regional unemployment rate	0.001 (0.002)	0.001 (0.002)	0.002 (0.002)	0.002* (0.001)	0.001 (0.002)	0.001 (0.002)	0.003 (0.002)	0.003* (0.001)
M: Eastern Germany	− 0.048 (0.052)	− 0.053 (0.052)	0.014 (0.010)	− 0.004 (0.014)	− 0.057 (0.049)	− 0.057 (0.049)	0.015 (0.011)	0.001 (0.019)
Population sample dummy		− 0.072*** (0.007)				− 0.050*** (0.006)		
N	21,999	21,999	9680	21,999	21,999	21,999	9680	21,999

(1) Full sample, unweighted, (2) full sample, unweighted with population sample dummy (3) population sample only, unweighted (4) full sample, weighted; M = individual average of covariate, S.E.: Standard errors clustered at the individual level in parantheses. ***/**/* denote statistical significance at the 0.001, 0.01, 0.05 levels, respectively. Source: PASS_0614_v1

**Table 13:** Average partial effects of all explanatory variables on the probability to be in a given state in t (PASS)

	Benefit receipt		Benefit receipt and employment		Inactivity		Employment	
	APE	S.E.	APE	S.E.	APE	S.E.	APE	S.E.
Benefit receipt in t−1	0.4228***	0.0184	0.0884***	0.0096	0.0240**	0.0093	− 0.5352***	0.0207
Benefit receipt and employment in t−1	0.1192***	0.0106	0.3484***	0.0181	− 0.0237***	0.0071	− 0.4439***	0.0194
Inactivity in t−1	0.1162***	0.0121	0.0022	0.0076	0.2752***	0.0204	− 0.3936***	0.0209
Benefit receipt in t0	0.0902***	0.0087	0.0027	0.0076	0.0306***	0.0067	− 0.1234***	0.0110
Benefit receipt and employment in t0	0.0214*	0.0086	0.0501***	0.0087	0.0006	0.0066	− 0.0721***	0.0105
Inactivity in t0	0.0556***	0.0092	− 0.0101	0.0086	0.0631***	0.0070	− 0.1086***	0.0103
Population sample	− 0.0330***	0.0065	− 0.0368***	0.0060	0.0025	0.0048	0.0672***	0.0075
Age (in years)	0.0001	0.0003	0.0013***	0.0002	− 0.0002	0.0002	− 0.0012***	0.0003
Male	0.0174***	0.0043	− 0.0126**	0.0040	− 0.0307***	0.0034	0.0258***	0.0048
Intermediate education	− 0.0258***	0.0045	− 0.0092*	0.0043	− 0.0050	0.0040	0.0400***	0.0056
High education	− 0.0424***	0.0071	− 0.0197**	0.0064	− 0.0077	0.0052	0.0698***	0.0078
Health restriction	0.0358***	0.0076	− 0.0186**	0.0068	0.0115	0.0071	− 0.0287**	0.0094
Migration Background	0.0010	0.0050	0.0086	0.0046	0.0075	0.0043	− 0.0172**	0.0058
Partner in same HH	− 0.0538***	0.0140	− 0.0087	0.0131	0.0092	0.0128	0.0533**	0.0176
Child < 2 years old	0.0450**	0.0147	− 0.0035	0.0140	0.0725***	0.0148	− 0.1140***	0.0153
Child 2–3 years old	0.0063	0.0132	0.0295*	0.0142	0.0047	0.0083	− 0.0405**	0.0124
Child 4–6 years old	− 0.0157	0.0114	0.0362**	0.0120	− 0.0046	0.0069	− 0.0159	0.0108
Child 7–16 years old	0.0049	0.0107	0.0282**	0.0102	− 0.0152*	0.0071	− 0.0178	0.0104
Regional unemployment rate	0.0029	0.0017	0.0026	0.0016	0.0013	0.0015	− 0.0067***	0.0020
Eastern Germany	0.0302	0.0631	0.0111	0.0352	− 0.0515	0.0347	0.0102	0.0570
M: Health restriction	0.0335***	0.0090	0.0047	0.0087	0.0076	0.0083	− 0.0458***	0.0115
M: Partner in same HH	0.0066	0.0137	− 0.0094	0.0132	0.0176	0.0129	− 0.0148	0.0171
M: Child < 2 years old	0.0170	0.0216	− 0.0080	0.0214	0.0074	0.0160	− 0.0164	0.0247
M: Child 2–3 years old	0.0111	0.0189	− 0.0063	0.0179	0.0001	0.0133	− 0.0049	0.0205
M: Child 4–6 years old	0.0221	0.0149	− 0.0323*	0.0136	0.0030	0.0107	0.0072	0.0154
M: Child 7–16 years old	− 0.0075	0.0116	− 0.0058	0.0106	0.0063	0.0085	0.0070	0.0118
M: Regional unemployment rate	0.0010	0.0017	− 0.0001	0.0015	− 0.0008	0.0015	− 0.0001	0.0020
M: Eastern Germany	− 0.0321	0.0609	− 0.0114	0.0343	0.0471	0.0399	− 0.0036	0.0583
Wave 4	0.0176*	0.0071	0.0188**	0.0067	− 0.0187**	0.0062	− 0.0177*	0.0082
Wave 5	0.0092	0.0067	0.0013	0.0061	− 0.0175**	0.0060	0.0070	0.0079
Wave 6	0.0103	0.0070	0.0040	0.0065	− 0.0210***	0.0061	0.0067	0.0079
Wave 7	0.0155*	0.0067	0.0056	0.0061	− 0.0147*	0.0061	− 0.0064	0.0077
Wave 8	0.0165*	0.0068	0.0111	0.0063	− 0.0194**	0.0061	− 0.0082	0.0079

N = 21,999; M = individual average of covariate, S.E.: Standard errors clustered at the individual level

***/**/* denote statistical significance at the 0.001, 0.01, 0.05 levels, respectively

Source: PASS_0614_v1

**Table 14:** Average partial effects of all explanatory variables on the probability to be in a given state in t (administrative data)

	Benefit receipt		Benefit receipt and employment		Inactivity		Employment	
	APE	S.E.	APE	S.E.	APE	S.E.	APE	S.E.
Benefit receipt in t−1	0.4543***	0.0201	0.1189***	0.0109	0.0077	0.0092	− 0.5810***	0.0220
Benefit receipt and employment in t−1	0.1307***	0.0105	0.3550***	0.0182	− 0.0361***	0.0066	− 0.4496***	0.0201
Inactivity in t−1	0.1021***	0.0126	0.0070	0.0082	0.3087***	0.0216	− 0.4178***	0.0217
Benefit receipt in t0	0.0892***	0.0104	0.0154	0.0091	0.0366***	0.0073	− 0.1412***	0.0131
Benefit receipt and employment in t0	0.0208*	0.0100	0.0683***	0.0100	0.0050	0.0066	− 0.0941***	0.0120
Inactivity in t0	0.0416***	0.0114	− 0.0093	0.0105	0.0654***	0.0073	− 0.0977***	0.0114
Population sample	− 0.0181**	0.0066	− 0.0309***	0.0067	0.0000	0.0047	0.0490***	0.0072
Age (in years)	− 0.0001	0.0003	0.0012***	0.0003	0.0001	0.0002	− 0.0012***	0.0003
Male	0.0161***	0.0043	− 0.0100*	0.0042	− 0.0281***	0.0033	0.0220***	0.0046
Intermediate education	− 0.0257***	0.0045	− 0.0012	0.0044	− 0.0025	0.0038	0.0293***	0.0052
High education	− 0.0391***	0.0074	− 0.0115	0.0069	− 0.0067	0.0051	0.0573***	0.0074
Health restriction	0.0393***	0.0076	− 0.0197**	0.0069	0.0086	0.0066	− 0.0282**	0.0087
Migration Background	− 0.0029	0.0049	0.0087	0.0047	0.0097*	0.0042	− 0.0155**	0.0056
Partner in same HH	− 0.0603***	0.0148	− 0.0188	0.0143	0.0174	0.0130	0.0617***	0.0180
Child < 2 years old	0.0487***	0.0145	− 0.0205	0.0138	0.0691***	0.0142	− 0.0973***	0.0151
Child 2–3 years old	0.0076	0.0130	0.0113	0.0139	0.0042	0.0079	− 0.0231*	0.0115
Child 4–6 years old	− 0.0092	0.0113	0.0258*	0.0122	− 0.0092	0.0065	− 0.0073	0.0101
Child 7–16 years old	0.0023	0.0112	0.0201	0.0109	− 0.0129	0.0069	− 0.0095	0.0103
Regional unemployment rate	0.0018	0.0017	0.0020	0.0017	0.0024	0.0014	− 0.0062**	0.0020
Eastern Germany	0.0045	0.0632	0.0579	0.0501	− 0.0400	0.0319	− 0.0223	0.0592
M: Health restriction	0.0239**	0.0090	− 0.0032	0.0089	0.0173*	0.0077	− 0.0380***	0.0107
M: Partner in same HH	0.0177	0.0142	0.0016	0.0141	0.0061	0.0130	− 0.0254	0.0169
M: Child < 2 years old	0.0008	0.0219	0.0035	0.0232	0.0164	0.0155	− 0.0206	0.0248
M: Child 2–3 years old	0.0089	0.0191	− 0.0055	0.0192	0.0024	0.0133	− 0.0058	0.0198
M: Child 4–6 years old	0.0188	0.0147	− 0.0190	0.0146	0.0049	0.0104	− 0.0047	0.0147
M: Child 7–16 years old	− 0.0058	0.0121	− 0.0023	0.0116	0.0033	0.0083	0.0048	0.0118
M: Regional unemployment rate	0.0018	0.0016	− 0.0005	0.0016	− 0.0019	0.0014	0.0006	0.0019
M: Eastern Germany	− 0.0061	0.0604	− 0.0548	0.0443	0.0320	0.0363	0.0289	0.0591
Wave 4	0.0060	0.0066	0.0148*	0.0065	− 0.0075	0.0056	− 0.0133	0.0075
Wave 5	− 0.0017	0.0067	− 0.0029	0.0065	− 0.0055	0.0058	0.0102	0.0076
Wave 6	0.0037	0.0071	0.0060	0.0069	− 0.0128*	0.0058	0.0030	0.0078
Wave 7	0.0080	0.0067	0.0046	0.0066	− 0.0058	0.0057	− 0.0068	0.0075
Wave 8	0.0058	0.0069	0.0028	0.0068	− 0.0078	0.0058	− 0.0008	0.0077

N = 21,999; M = individual average of covariate, S.E.: Standard errors clustered at the individual level

***/**/* denote statistical significance at the 0.001, 0.01, 0.05 levels, respectively

Source: PASS_0614_v1 & LHG V08,01,00 – 201504

**Table 15:** Average partial effects of all explanatory variables on the probability to be in a given state in t (PASS including individuals without consent to data linkage or not found in administrative data)

	Benefit receipt		Benefit receipt and employment		Inactivity		Employment	
	APE	S.E.	APE	S.E.	APE	S.E.	APE	S.E.
Benefit receipt in t−1	0.4005***	0.0165	0.0827***	0.0087	0.0305***	0.0088	− 0.5136***	0.0188
Benefit receipt and employment in t−1	0.1144***	0.0095	0.3184***	0.0160	− 0.0233***	0.0065	− 0.4096***	0.0176
Inactivity in t−1	0.1140***	0.0104	0.0040	0.0068	0.2615***	0.0173	− 0.3796***	0.0180
Benefit receipt in t0	0.0881***	0.0078	0.0047	0.0068	0.0322***	0.0063	− 0.1250***	0.0103
Benefit receipt and employment in t0	0.0210**	0.0077	0.0481***	0.0078	− 0.0039	0.0060	− 0.0652***	0.0097
Inactivity in t0	0.0532***	0.0083	− 0.0104	0.0075	0.0654***	0.0065	− 0.1081***	0.0094
Population sample	− 0.0365***	0.0058	− 0.0426***	0.0052	0.0059	0.0043	0.0732***	0.0068
Age (in years)	0.0002	0.0002	0.0012***	0.0002	0.0000	0.0002	− 0.0014***	0.0003
Male	0.0161***	0.0038	− 0.0119***	0.0035	− 0.0360***	0.0031	0.0318***	0.0044
Intermediate education	− 0.0256***	0.0040	− 0.0069	0.0038	− 0.0090*	0.0037	0.0415***	0.0051
High education	− 0.0417***	0.0061	− 0.0225***	0.0055	− 0.0115*	0.0047	0.0757***	0.0069
Health restriction	0.0362***	0.0067	− 0.0194**	0.0061	0.0135*	0.0065	− 0.0303***	0.0085
Migration Background	0.0058	0.0044	0.0104*	0.0040	0.0080*	0.0039	− 0.0241***	0.0053
Partner in same HH	− 0.0489***	0.0122	− 0.0093	0.0113	0.0154	0.0112	0.0428**	0.0151
Child < 2 years old	0.0382**	0.0127	− 0.0013	0.0125	0.0758***	0.0140	− 0.1127***	0.0145
Child 2–3 years old	0.0074	0.0113	0.0209	0.0121	0.0052	0.0078	− 0.0334**	0.0115
Child 4–6 years old	− 0.0122	0.0097	0.0268*	0.0104	− 0.0050	0.0065	− 0.0096	0.0100
Child 7–16 years old	0.0032	0.0093	0.0204*	0.0092	− 0.0151*	0.0065	− 0.0084	0.0098
Regional unemployment rate	0.0033**	0.0012	0.0011	0.0013	0.0014	0.0012	− 0.0059***	0.0014
Eastern Germany	0.0494	0.0419	− 0.0271	0.0316	− 0.0154	0.0340	− 0.0068	0.0504
M: Health restriction	0.0373***	0.0079	0.0053	0.0077	0.0021	0.0074	− 0.0447***	0.0102
M: Partner in same HH	0.0014	0.0120	− 0.0064	0.0115	0.0117	0.0115	− 0.0067	0.0151
M: Child < 2 years old	0.0387*	0.0181	− 0.0188	0.0184	0.0050	0.0145	− 0.0249	0.0222
M: Child 2–3 years old	0.0062	0.0159	0.0020	0.0154	0.0019	0.0116	− 0.0101	0.0182
M: Child 4–6 years old	0.0178	0.0127	− 0.0247*	0.0122	0.0045	0.0096	0.0024	0.0143
M: Child 7–16 years old	− 0.0016	0.0101	0.0009	0.0097	0.0085	0.0077	− 0.0077	0.0111
M: Regional unemployment rate	0.0006	0.0013	0.0012	0.0013	− 0.0009	0.0012	− 0.0010	0.0015
M: Eastern Germany	− 0.0495	0.0408	0.0312	0.0337	0.0052	0.0367	0.0131	0.0516
Wave 4	0.0177**	0.0061	0.0179**	0.0059	− 0.0195***	0.0054	− 0.0161*	0.0072
Wave 5	0.0086	0.0059	0.0005	0.0054	− 0.0185***	0.0053	0.0093	0.0070
Wave 6	0.0122*	0.0060	0.0015	0.0055	− 0.0152**	0.0054	0.0016	0.0068
Wave 7	0.0123*	0.0058	0.0038	0.0053	− 0.0133*	0.0053	− 0.0027	0.0066
Wave 8	0.0142*	0.0059	0.0083	0.0056	− 0.0186***	0.0053	− 0.0039	0.0069

N = 28,034; M = individual average of covariate, S.E.: Standard errors clustered at the individual level

***/**/* denote statistical significance at the 0.001, 0.01, 0.05 levels, respectively

Source: PASS_0614_v1

## Footnotes

1 Not only government benefits can be subject to measurement error but also income information. Pavlopoulos et al. (2012) study the effect of measurement error in income information on low-pay transition. They do not use additional (correct) data to study measurement error but a Mixed Latent Markov model with two measurements of income. They find half of the observed transitions to be caused by measurement error.

2 The PASS offers combined weights to balance the two subsamples. As we are not primarily interested in determining the absolute level of state dependence in German welfare benefit receipt, but in determining the role of misreporting for the estimated state dependence, we present only unweighted descriptive and estimation results. In the estimations, we control for the two subsamples with a dummy variable.

3 We restricted our analyses to observations younger than 58 years because of the special position of individuals aged 58 years or above in the German welfare benefit system: Since 2008 welfare recipients are no longer counted as unemployed after receiving welfare benefit for at least 12 months after their 58th birthday without an offer of work that is liable to social insurance.

4 In our observation window, marginal employment was defined as employment with monthly earnings up to €400 through 2012 and up to €450 since 2013. For marginal employment, the employee is not required to pay social security contributions.

5 We include education according to the Casmin classification (König et al. 1988).

6 The average partial effects are obtained from the coefficient estimates shown in Appendix: Tables 10 (PASS) and 11 (Administrative data). The average partial effects must always be interpreted relative to the base category, which is ‘employed in t−1’. The average partial effects for all variables used in the estimation are shown in Appendix: Tables 13 (PASS) and 14 (Administrative data).

7 To address the potential selectivity of the data linkage, we rerun the estimation with survey data including the individuals who could not be linked to the administrative data. It turned out that our main findings are robust to this data selection (see Appendix: Table 15 ).
